# An East Coast Convalescent Home

**Published:** 1923-09

**Authors:** 


### AN EAST COAST CONVALESCENT HOME.

THE Board of Management of the East Suffolk and Ipswich Hospital is to be congratulated on its scheme for a new Convalescent Home in connection with that Institution at Felixstowe. The new building promises to be one of the best of its kind in the Kingdom.

### A Noble Bequest.

A former President of the hospital—the late Dr. J. H. Bartlet—left a large residuary estate to the Board of Management for building, equipping and maintaining a Convalescent Home for the benefit only of patients from the hospital. Although the testator has been dead for over six years, the estate, owing to its magnitude and complications, has not yet been finally wound up, but the Court has already sanctioned the transfer to the hospital of the bulk of the funds forming the residuary estate, and from the balance sheet for the year ended May 31, 1923, it appears that the total amount available for the Convalescent Home, including interest accrued, etc., amounts to £229,869 11s. 6d. Of this sum £32,152 13s. has been expended in the purchase of a site at Felixstowe, and the erection thereon of the quarters for the nursing and domestic staffs. The High Court has now sanctioned the acceptance of a tender for the construction of the Home itself at a cost of approximately £50,000. The plans have been prepared by Mr. H. Munro Cautley, A.R.I.B.A., the well-known institutional architect, and the building, which has just been begun, is due for completion in the spring of 1925. In the meantime and for the past five years a temporary Convalescent Home has been maintained out of the funds of the bequest.

### A Unique Scheme.

The financial management of the scheme is in the hands of Mr. Arthur Griffiths, O.B.E., the Secretary of the hospital, who has been able to accumulate a considerable sum from the dividend income available for the temporary Home, and this is included in the total amount referred to. This Convalescent Home scheme is unique in that it not only provides free treatment for the patients from the hospital, but includes free transport to and from the hospital —a distance of twelve miles. Mr. Griffiths anticipates that when the expenditure for building, equipment and furnishing has been defrayed, there will still remain a sum of £140,000 available for the endowment.

